# Non-coding RNAs as Regulators of Lymphangiogenesis in Lymphatic Development, Inflammation, and Cancer Metastasis

**DOI:** 10.3389/fonc.2019.00916

**Published:** 2019-09-20

**Authors:** Ming-xin Cao, Ya-ling Tang, Wei-long Zhang, Ya-Jie Tang, Xin-hua Liang

**Affiliations:** ^1^State Key Laboratory of Oral Diseases & National Clinical Research Center for Oral Diseases, Department of Oral and Maxillofacial Surgery, West China Hospital of Stomatology, Sichuan University, Chengdu, China; ^2^State Key Laboratory of Oral Diseases & National Clinical Research Center for Oral Diseases, Department of Oral Pathology, West China Hospital of Stomatology, Sichuan University, Chengdu, China; ^3^State Key Laboratory of Microbial Technology, Shandong University, Qingdao, China; ^4^Hubei Key Laboratory of Industrial Microbiology, Key Laboratory of Fermentation Engineering (Ministry of Education), Hubei Provincial Cooperative Innovation Center of Industrial Fermentation, Hubei University of Technology, Wuhan, China

**Keywords:** non-coding RNA (ncRNA), lymphangiogenesis, lymphatic development, inflammation, cancer metastasis

## Abstract

Non-coding RNAs (ncRNAs), which do not encode proteins, have pivotal roles in manipulating gene expression in development, physiology, and pathology. Emerging data have shown that ncRNAs can regulate lymphangiogenesis, which refers to lymphatics deriving from preexisting vessels, becomes established during embryogenesis, and has a close relationship with pathological conditions such as lymphatic developmental diseases, inflammation, and cancer. This review summarizes the molecular mechanisms of lymphangiogenesis in lymphatic development, inflammation and cancer metastasis, and discusses ncRNAs' regulatory effects on them. Therapeutic targets with regard to lymphangiogenesis are also discussed.

## Introduction

Lymphangiogenesis are termed lymphatics deriving from preexisting vessels, and generally progress through a number of stages: establishment of lymphatic endothelial cell (LEC) identity, formation of the primary lymphatic structures, maturation, and remodeling of the lymphatic vessels (1, 2). The normal growth and development of lymphatics contribute to their non-negligible roles in the cardiovascular system, including maintaining tissue fluid homeostasis, directing the trafficking of immune cells during immunosurveillance, and absorbing dietary lipids from the digestive tract (3, 4). However, in developmental diseases such as lymphedema, lymphangiectasia, and vascular malformations, or in inflammatory conditions such as infectious diseases, and after surgical intervention, lymphatic function is impaired which might lead to swellings and edema (5). In other pathological conditions, such as cancer, it might be essential to inhibit lymphangiogenesis, thus preventing cancer metastasis (6). Our comprehension of lymphangiogenesis regulation is mainly based on understanding the functions of proteins and their interactions, while it is widely known that only 3–5% of our genome encodes proteins and protein-target therapy may cause drug resistance (7). Therefore, the clinical regulation of lymphangiogenesis further requires other types of targets for successful intervention.

Currently, emerging studies have implicated connections between lymphangiogenesis and non-coding RNAs (ncRNAs), especially the well-known microRNAs (miRNAs), long non-coding RNAs (lncRNAs), and the newly discovered circular RNAs (circRNAs), which are transcribed from the vast majority of human genome. ncRNAs, though most do not encode proteins, contain genetic information or have functions in the biological process of cells. ncRNAs include structural RNAs such as tRNAs and rRNAs, which are abundant and have well-defined roles in translation, and regulatory RNAs such as miRNAs, lncRNAs, and circRNAs. These regulatory RNAs contain physiological and pathological functions, by altering protein expressions, interacting as signaling partners with specific proteins or acting as scaffolds for protein complexes to change signaling pathways (8, 9). Preclinical studies and increased success rates of ncRNA-target therapy provide a possibility of targeting ncRNAs in lymphangiogenesis-related disorders (10, 11).

Just as the old Chinese saying goes that “one stone with three birds,” understanding the underlying mechanisms important for ncRNAs targeting lymphangiogenesis in lymphatic developmental diseases, inflammation and cancer metastasis will help build new therapeutics when more than one disorder exists. Here, we review the molecular mechanisms of lymphangiogenesis in lymphatic development, inflammation and cancer metastasis, emphasize the ncRNAs' regulation on them, and hope to harness this knowledge for translational medicine.

## Lymphatic Development

### Lymphatic Development

In vertebrates, the first definitive sign of lymphatic development is the presence of endothelial cells with the expression of PROX1, considered to be the master regulator of LEC fate specification in cardinal veins (12, 13). *Prox1* deletion in mice led to a complete absence of the lymphatic vasculature. PROX1-positive LECs bud, sprout and migrate away from both the cardinal and intersomitic veins to form the primary lymphatic plexus and sacs (14–16). Once exiting the veins, primitive LECs exhibit LEC identity including podoplanin, and increased levels of VEGFR-3 and NRP2 (14–16). This exiting process is absolutely dependent on VEGF-C, which acts via its tyrosine kinase receptor VEGFR-3 and the non-signaling co-receptor NRP2 (16–18). PROX1-positive LECs do not egress from the veins in embryos when deficient in key regulators of VEGF-C/VEGFR-3 signaling, such as collagen and calcium binding EGF domains 1 (CCBE1) (16, 19, 20). As for the initiation of PROX1 expression in venous endothelial cells, transcription factor SOX18 (21) and NR2F2/COUP-TFII (22) are thought to be critical. Intriguingly, PROX1 expression is induced in the cells of dorsolateral aspect of veins, while its inducer/co-regulator, SOX18, and COUP-TFII, are expressed throughout the cardinal vein endothelial cells (12, 23). Current explanation for this polarization involves retinoic acid signaling (24), Notch signaling (25) and BMP2 signaling pathway (26). They are all researched to regulate the emergence of lymphatic endothelial progenitor cells from the veins.

After LECs migrating and forming primary lymphatic vascular structures, major events involved in lymphatic development includes formation of lympho-venous valves, induction of platelet aggregation in valve regions, and remodeling of the initial lymphatic plexus to form a hierarchical lymphatic vascular tree (27, 28). Upregulation of FOXC2, together with high levels of PROX1 and GATA2, exist in the clusters of cells destined to form valves (29, 30). FOXC2 and PROX1 coordinately control expression of the gap junction protein connexin37 and activation of calcineurin/NFAT signaling, which are required for the assembly and delimitation of lymphatic valve territory during development (31). And cell surface molecules including the planar cell polarity pathway members, CELSR1 and VANGL2 (32), signaling pathways including Notch (33), BMP (34), and Semaphorin/Neuropilin/Plexin axes (35), and mechanical stimuli including disturbed flow are also important for valve development (31). As for platelet aggregation, signaling induced by podoplanin on the surface of LECs can bind to platelet C-type lectin-like receptor2 (CLEC2) to prevent blood entering into the lymphatics (27, 36). Both valves and platelet thrombi are crucial for separating the blood and lymphatic vascular compartments. In addition, signaling pathways such as angiopoietin/Tie signaling (37, 38), EphrinB2 signaling (39), and Reelin signaling (40), are significant for primitive lymphatic plexus remodeling to form initial and collecting vessels.

### miRNAs and Lymphatic Development

miRNAs (19–24 nucleotides) are endogenous, non-protein-coding small RNAs that serve as post-transcriptional gene regulators (41, 42). According to the miRBase (version 21.0), over 60% of the protein-coding genes in human are targeted by miRNAs (43). Recent studies have defined the critical roles of miRNAs in lymphangiogenesis. Kazenwadel et al. demonstrated that miR-181a, the first discovered miRNA that targets PROX1, could bind to the Prox1 3′-UTR, resulting in translational inhibition and transcript degradation (44). Increased miR-181a in primary embryonic LECs led to the substantial reduced levels of PROX1 and resulted in reversion of LEC identity toward a blood vascular phenotype. Inhibition of endogenous miR-181a in blood endothelial cells (BECs) leads to elevated PROX1 expression, therefore promoting the acquisition of LEC identity. miR-31, as a novel BEC-specific miRNA, inhibited lymphatic lineage-specific differentiation in BECs, at least in part by repressing PROX1 *in vitro*, and impaired lymphatic development and venous sprouting *in vivo* (45). miR-31 candidate targets also include FOXC2, which is required for specification of lymphatic capillaries vs. collecting lymphatic vessels (46, 47), and RAMP2, which triggers lymphangiogenesis in response to adrenomedullin signaling (48). Recent evidence has shown that BMP2 signaling, the negative modulator for lymphatic fate during development, could promote both miR-181a and miR-31 in a SMAD-dependent manner, thus negatively regulating PROX1 expression at the post-transcriptional level. BMP2 signaling is therefore essential for constructing therapeutic manipulation of lymphangiogenesis in development (26).

**Figure 1:**
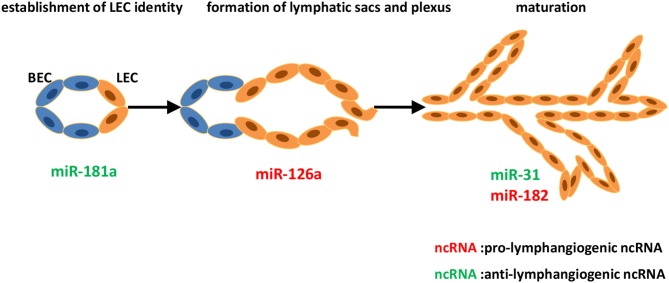
Identified ncRNAs regulating lymphangiogenesis in lymphatic development. The development of lymphatic vascular network starts with the cells of cardinal vein losing blood endothelial characteristics and acquiring lymphatic endothelial cell (LEC) identity. LECs then bud off the cardinal vein and form lymphatic sacs and plexus. Subsequently, remodeling of the primitive lymphatic vasculature begins, and becoming a hierarchical network. We described ncRNAs, mostly miRNAs identified to-date, which influence different steps of developmental lymphangiogenesis.

miR-182, which is induced by JunB and attenuates FoxO1 expression in zebrafish, is crucial for the formation of parachordal lymphangioblasts and the thoracic duct. This JunB/miR-182/FoxO1 axis is regarded as a novel key player in governing lymphangiogenesis (49). A recent study has identified miR-126a as a director of LEC migration and lymphatic assembly. *In vivo* studies by Chen et al. reported that VEGF-C/FLT4 signaling acted as a cooperator of miR-126a, allowing modulation of lymphangiogenic sprout formation. miR-126a upregulated CXCL12a by targeting its 5′-UTR, then inducing chemokine signaling, resulting in parachordal lymphangioblast extension along a horizontal myoseptum (50, 51). Subsequent research confirmed miR-126 as a conserved modulator of lymphatic development *in vivo* and *in vitro*, and put forward the potential of miR-126 in preventing lymphedema, the most recognized aspect of lymphatic system malfunction as a result of genetic cause (52) (Figure 1).

## Inflammation

### Lymphangiogenesis in Inflammation

Inflammation is a common feature of various conditions, characterized by pathological neovascularization, including hemangiogenesis and lymphangiogenesis. Hemangiogenesis refers to the new outgrowth from pre-existing blood vessels, and is an important pathological aspect of chronic inflammatory diseases (53). Lymphangiogenesis in inflammation is often induced by factors produced by macrophages and dendritic cells, and its existence involves tissue edema reduction, immune response initiation, and antigen clearance (54). Macrophages, especially CD11b^+^ macrophages, play a pivotal role in the inflammatory lymphangiogenesis mediated by VEGF ligands (55, 56). The VEGF family consists of five members that bind to and activate three distinct receptors. VEGF-A binds to VEGFR-1 and VEGFR-2; placental growth factor (PlGF) and VEGF-B bind only to VEGFR-1; and VEGF-C and VEGF-D bind to VEGFR-2 and VEGFR-3. Generally speaking, ligation of VEGF-A to VEGFR-2 induces only hemangiogenesis, while interactions of VEGF-C/D and VEGFR-3 mediate lymphangiogenesis (57–59). However, recent observations contradicted this notion and found that there was some crosstalk between them. VEGF-C produced by activated macrophages can induce local proliferating and sprouting of preexisting lymphatic cells (60, 61), while VEGF-A, expressed by activated leucocytes in inflammatory context, can recruit VEGFR-1-expressing macrophages, which are known to release VEGF-C/D, thus inducing inflammatory lymphangiogenesis (62). Maruyama et al. showed that VEGFR-3-expressing CD11b^+^ macrophages could directly transdifferentiate into lymphatic endothelial cells (LECs), forming cell aggregates that gradually developed into sprouting lymphatic vessels (63). In addition to the two ways of macrophages supporting lymphangiogenesis, dendritic cells activated by IL-1β and TNF-α in inflammation milieu can also migrate to lymphatic vessels, express VEGF-C, promoting lymphangiogenesis (64).

### miRNAs and Inflammatory Lymphangiogenesis

The first example of the regulatory role of miRNA in inflammatory lymphangiogenesis is miR-1236, which is expressed in LECs and binds to VEGFR-3. Jones et al. demonstrated that miR-1236, induced by IL-1β, could negatively regulate VEGFR-3 expression and VEGFR-3-dependent signaling Akt and ERK1/2, and attenuate VEGF-C/VEGFR-3 mediated LECs migration and tube formation in primary human LECs *in vitro*. They also found that miR-1236 could reduce lymphangiogenesis *in vivo* (65, 66). Considering the fact that IL-1β contributes to initial lymphangiogenesis by inducing VEGFs and also induces miR-1236, which in turn suppresses VEGFR-3-dependent signaling, modulation of VEGFR-3 levels using miR-1236 may be a promising approach for the treatment of inflammatory diseases. Additionally, studies by Chakraborty et al. revealed that miR-9 expressed on inflamed LECs, which was induced by TNF-α, could inhibit NF-κB-mediated inflammation, increase VEGFR-3 and induce LEC proliferation and tube formation to activate VEGFR-3-mediated lymphangiogenesis (67). Studies concerning inflammatory lymphangiogenesis usually involved models of corneal lymphangiogenesis, as cornea exhibited alymphatic feature under normal condition and lymphangiogenesis under pathologic insults such as inflammation. miR-466, miR-184, and miR-199a/b-5p have all been reported to be significantly downregulated in corneal inflammatory lymphangiogenesis, and accordingly, inhibit corneal lymphatic growth *in vivo* and suppress LECs functions of adhesion, migration, and tube formation *in vitro*. This offers a clinical potential for lymphangiogenesis interference and lymphatic-related diseases treatment (68–70). Wang et al. revealed that miR-132 isolated from exosomes of VEGF-C-treated adipose-derived stem cells could be directly transferred to LECs and promote LECs proliferation, migration, and tube formation by targeting Smad-7 and activating TGF-β/Smad signaling, thus reversing acute to chronic inflammatory processes in inflammatory bowel disease (71).

**Figure 2:**
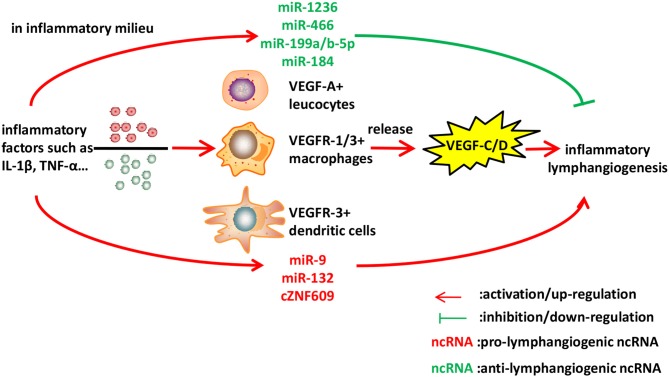
Identified ncRNAs regulating lymphangiogenesis in inflammation. Inflammatory cytokines such as IL-1β and TNF-α, stimulate macrophages, leucocytes, and dendritic cells to express VEGF-C/D, leading to inflammatory lymphangiogenesis. We described ncRNAs, mostly miRNAs identified to-date, which influence inflammatory lymphangiogenesis.

Recently, circular RNA (circRNA) cZNF609 could serve as a sponge for miR-184 and subsequently elevated miR-184-target gene heparanase in inflamed corneas, which could significantly elevate VEGF-C expression and facilitate lymphangiogenesis *in vitro* and *in vivo*. However, whether cZNF609 intervention could act as a therapeutic strategy in preventing inflammation-induced lymphangiogenesis and treating ocular inflammatory diseases remains unknown, therefore requires further investigation (72) (Figure 2).

## Cancer Metastasis

### Lymphangiogenesis in Tumor

Cancer metastasis, the dissemination of cancer cells from primary tumors to distant organs, is considered to be the primary cause of cancer-related deaths. The majority of epithelial cancers firstly develop metastasis through spreading via lymphatic vessels (73). Tumor hypoxia microenvironment stimulates tumor cells, tumor stroma cells, and tumor-infiltrating inflammatory cells to express a series of lymphangiogenic factors, including the well-known VEGF family, especially VEGF-A/C/D (74), and other mediators such as PDGF-BB (75), IGF1/2 (76), FGF2 (77–79), HGF (80, 81), angiopoietin-2 (82), sphingosine-1-phosphate (83), adrenomedullin (84), and IL-7 (85, 86). In response to these factors, lymphangiogenesis can start from existing lymphatic vessels via sprouting, LEC proliferation, and formation of intra- and peri-tumoral lymphatics. Additionally, it can also derive from precursor LEC and bone marrow-derived cells (87, 88). After disseminating into sentinel lymph nodes (SLNs, the first tumor draining LN), lymphangiogenic factors induce LN lymphangiogenesis prior to the arrival of cancer cells. Besides inducing new lymphatic vessels, tumors can co-opt existing lymphatics at the primary site (73).

Crosstalk between tumors and lymphatic vessels are bidirectional. In addition to being influenced by tumors mentioned above, lymphatic vessels in return can contribute to cancer metastasis by secreting chemokines CCL21 (89) or CXCL12 (90), which bind to CCR7 or CXCR4 receptors, respectively, expressed in invading cancer cells, thus recruiting cancer cells toward lymphatic vessels. Lymphatic vessels can also provide a cancer stem cell niche (91) and modulate antitumor immune responses (92, 93), affecting metastatic tumor cells.

### miRNAs and Lymphatic Metastasis

The most established lymphangiogenic factor, VEGF-C, can be targeted by miR-128 in human non-small cell lung cancer (NSCLC) cells and human umbilical vein endothelial cells (HUVECs). Hu et al. demonstrated that miR-128 could directly suppress VEGF-C and simultaneously decrease VEGF-A, VEGFR-2, and VEGFR-3 indirectly, thus reducing the phosphorylation of downstream VEGFR signaling pathways extracellular signal-regulated kinase (ERK1/2), phosphatidylinositol 3-kinase(AKT), and p38, resulting in tube formation inhibition *in vitro*. Furthermore, by analyzing immunohistochemical staining with anti-LYVE-1 antibodies of tumor tissues, they found out that miR-128 could suppress lymphangiogenesis of tumor xenografts *in vivo*, suggesting the therapeutic significance of miR-128 in NSCLC (94). VEGF-C can also be indirectly targeted by miR-206. Keklikoglou et al. revealed that, in pancreatic adenocarcinoma, miR-206 suppressed lymphangiogenesis through abrogating the expression of VEGF-C. Also, there was a striking reduction in the number of capillary-like tubes and intratumoral lymphatics coverage in the existence of miR-206, indicating that miR-206-based therapy might have important translational implications in pancreatic adenocarcinoma treatment (95). In chondrosarcoma, a series of studies have indicated that miR-381, miR-507, miR-27b, miR-624-3p, and miR-186 contributed to the inhibition of VEGF-C-dependent lymphangiogenesis with different mechanisms, all of which provided information on the potential miRNA-based molecular diagnosis and treatment for VEGF-C-mediated lymphangiogenesis in chondrosarcoma (96–100). In oral squamous cell carcinoma (OSCC) cells, Lin et al. found that decreased miR-300, which was suppressed by WNT1-inducible signaling pathway protein-1 (WISP-1), could contribute to VEGF-C-dependent lymphangiogenesis (101). And inhibited miR-195-3p, targeted by chemokine CCL4, could also induce VEGF-C and lymphangiogenesis in OSCC cells (102).

Besides VEGF-C, another member of VEGF family, VEGF-A, could induce lymphangiogenesis apart from angiogenesis, and accelerate nodal metastasis in OSCC (103). Research showed that miR-126 negatively regulated VEGF-A, and thus decreased lymphatic vessel density in OSCC specimens (104). Neuropilin-2 (NRP2), another important regulator of lymphangiogenesis, was directly suppressed by miR-486-5p in colorectal carcinoma cells, leading to the reduction of peritumoral lymphatic microvessels *in vivo*, and thus demonstrating the suppressor role of miR-486-5p in colorectal carcinoma (105). miR-93 was reported to inhibit angiogenesis and lymphangiogenesis by targeting angiopoietin2, and thus suppressed malignant pleural effusion, a sign of an advanced tumor stage (106).

Conversely, there were also pro-lymphangiogenetic miRNAs in cancer metastasis. miR-7 in gastric cancer cells promoted p65-mediated aberrant NF-κB activation and its downstream metastasis-related molecules including VEGF-C, and thus facilitated metastasis by alleviating hemangiogenesis, lymphangiogenesis, and inflammatory cells infiltration (107). miR-548k acted as a pro-lymphangiogenic miRNA in esophageal squamous cell carcinoma (ESCC) via promoting VEGF-C secretion and stimulating lymphangiogenesis, highlighting its crucial role as a new diagnostic and prognostic marker of ESCC (108). miR-27a could be induced in LECs by co-culturing with colon cancer cells, and promoted lymphangiogenesis via targeting SMAD4, a pivotal member of the TGF-β signaling and a tumor suppressor (109). Additionally, exosomes secreted from cancer cells could mediate lymphangiogenesis. A recent study showed that cervical squamous cell carcinoma (CSCC)-secreted exosomal miR-221-3p could transfer into LECs to promote lymphangiogenesis and lymphatic metastasis through downregulation of VASH1, representing a novel diagnostic biomarker and therapeutic target for metastatic CSCC patients in early stage (110). Furthermore, circulating miR-10b and miR-373 were shown to be potential biomarkers in detecting lymph node metastasis of breast cancer (111). There have been some studies focusing on miRNAs associated with lymphangiogenesis in various cancers, such as gastric cancer (112), lung cancer (113), and papillary thyroid cancer, of which the underlying mechanisms need to be further elucidated (114, 115).

### LncRNAs and Lymphatic Metastasis

Although the functional roles of miRNAs in lymphangiogenesis are now established, relatively less is known about the regulatory roles of lncRNAs (>200 nts) (116, 117). Known as the “transcriptional noise,” lncRNAs rarely code for proteins, but are regulated like that of protein coding RNAs, being subjected to transcriptional regulation or even splicing (118, 119). Unlike miRNAs acting mainly as post-transcriptional repressors, functional lncRNAs can regulate gene expression at various levels, such as chromatin modification, transcriptional and post-transcriptional processing (120, 121). A number of findings have indicated the contribution of lncRNAs in cancer metastasis (122), while the question as to whether these lncRNAs are involved in lymphangiogenesis and lymph node metastasis is still being studied.

**Figure 3:**
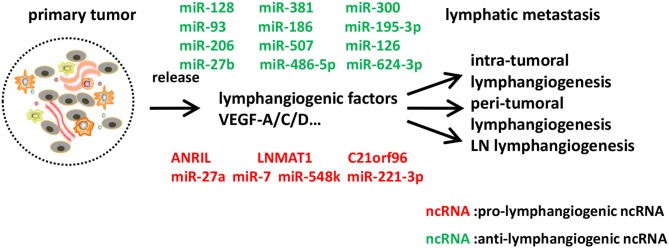
Identified ncRNAs regulating lymphangiogenesis in cancer metastasis. In tumor microenvironment, tumor cells, tumor stroma cells, and tumor-infiltrating inflammatory cells express a series of lymphangiogenic factors, and thus lead to tumor lymphangiogenesis and lymphatic metastasis. We described ncRNAs, mostly miRNAs identified to-date, which influence different steps of lymphangiogenesis in cancer metastasis.

Recent evidence has shown that antisense non-coding RNA in the INK4 locus (ANRIL), a kind of lncRNA, induced lymphangiogenesis and lymphatic metastasis in colorectal cancer. ANRIL expression was correlated with the increased expressions of VEGF-C, VEGFR3, LYVE-1, and tube formation in both colorectal cancer cell lines and surgical specimens. ANRIL downregulation reduced lymphatic metastasis rate, lymphatic microvessel density (LMVD), and the expressions of VEGF-C, VEGFR3, LYVE-1, representing the potential role of ANRIL as a therapeutic target in colorectal cancer (123). In addition, a lncRNA termed Lymph Node Metastasis Associated Transcript 1 (LNMAT1), upregulated in lymph node-positive bladder cancer and associated with lymph node metastasis and prognosis, could epigenetically activate CCL2 expression and recruit macrophages into the tumor, which promoted lymphangiogenesis via VEGF-C secretion. LNMAT1 may represent a potential therapeutic target for clinical intervention in lymph node-metastatic bladder cancer (124). Other lymphangiogenesis-related lncRNAs still need further functional studies to verify their roles. For example, C21orF96 was overexpressed in positive lymph node and gastric cancer tissues, and promoted tubular formation in gastric cancer cell lines, while its pathogenesis was less well-characterized (125). MALAT-1 (126), UCA1 (127), HOTTIP (128), and HOTAIR (129) have all been proven to be associated with lymph node metastasis in various cancers and might serve as novel predictors, but well-designed studies are awaited to explain the mechanisms underlying it for uncovering better therapeutic strategies (Figure 3).

## Conclusions and Future Perspectives

In summary, ncRNAs, the dark matter of the genome, account for >80% of total mature RNA and have many crucial, but as yet, undefined roles in regulating lymphangiogenesis concerning lymphatic developmental disorders, inflammatory diseases, and cancer metastasis (130). The two major types of regulatory ncRNAs, miRNAs, and lncRNAs, modulate inter-related steps and mediators of lymphangiogenesis, therefore exert their influence on lymphatic developmental disorders, inflammatory diseases or cancer metastasis, if not all of them. The identification of key pro-lymphangiogenic and anti-lymphangiogenic ncRNAs is currently the aim of investigation and will underpin the generation of novel therapeutic targets, as well as potential targets on diagnosis, prognosis and response prediction (Table 1).

**Table 1:** ncRNAs that mediate lymphangiogenesis in lymphatic development, inflammation, and cancer metastasis.

**ncRNAs**	**Micro-environment**	**Lymphangiogenetic function**	**Mechanisms of action**	**References**
miR-181a	Lymphatic development	Anti-lymphangiogenesis	Inhibits PROX1	(44)
miR-31		Anti-lymphangiogenesis	Inhibits PROX1, FOXC2, and RAMP2	(45)
miR-182		Pro-lymphangiogenesis	Induced by JunB and inhibits FoxO1	(49)
miR-126a		Pro-lymphangiogenesis	Cooperated with VEGF-C/FLT4 signaling and enhances CXCL12a	(50–52)
miR-1236	Inflammation	Anti-lymphangiogenesis	Induced by IL-1β and inhibits VEGFR-3	(65, 66)
miR-9		Pro-lymphangiogenesis	Induced by TNF-α and increases VEGFR-3	(67)
miR-466		Anti-lymphangiogenesis	Inhibits PROX1	(70)
miR-184		Anti-lymphangiogenesis	Needs further investigation	(69)
miR-199a/b-5p		Anti-lymphangiogenesis	Inhibits DDR1	(68)
miR-132		Pro-lymphangiogenesis	Inhibits Smad-7 and activates TGF-β/Smad signaling	(71)
cZNF609		Pro-lymphangiogenesis	Elevates heparanase by sponging miR-184	(72)
miR-128	NSCLC	Anti-lymphangiogenesis	Inhibits VEGF-C directly	(94)
miR-206	Pancreatic adenocarcinoma	Anti-lymphangiogenesis	Inhibits VEGF-C indirectly	(95)
miR-381	Chondrosarcoma	Anti-lymphangiogenesis	Inhibits VEGF-C directly	(99)
miR-507	Chondrosarcoma	Anti-lymphangiogenesis	Inhibits VEGF-C directly	(98)
miR-27b	Chondrosarcoma	Anti-lymphangiogenesis	Inhibits VEGF-C directly	(97)
miR-624-3p	Chondrosarcoma	Anti-lymphangiogenesis	Inhibits VEGF-C directly	(96)
miR-186	Chondrosarcoma	Anti-lymphangiogenesis	Inhibits VEGF-C directly	(100)
miR-300	OSCC	Anti-lymphangiogenesis	Suppressed by WISP-1 and decreases VEGF-C expression	(101)
miR-195-3p	OSCC	Anti-lymphangiogenesis	Suppressed by CCL4 and decreases VEGF-C expression	(102)
miR-126	OSCC	Anti-lymphangiogenesis	Inhibits VEGF-A	(104)
miR-486-5p	Colorectal cancer	Anti-lymphangiogenesis	Inhibits NRP2 directly	(105)
miR-93	Malignant pleural effusion	Anti-lymphangiogenesis	Inhibits angiopoietin2 directly	(106)
miR-7	Gastric cancer	Pro-lymphangiogenesis	Promotes VEGF-C expression	(107)
miR-548k	ESCC	Pro-lymphangiogenesis	Promotes VEGF-C secretion	(108)
miR-27a	Colon cancer	Pro-lymphangiogenesis	Inhibits SMAD4	(109)
miR-221-3p	CSCC	Pro-lymphangiogenesis	Inhibits VASH1	(110)
ANRIL	Colorectal cancer	Pro-lymphangiogenesis	Correlates with increased VEGF-C, VEGFR-3, LYVE-1	(123)
LNMAT1	Bladder cancer	Pro-lymphangiogenesis	Increases CCL2 and recruits macrophage to secret VEGF-C	(124)
C21orF96	Gastric cancer	Pro-lymphangiogenesis	Needs further investigation	(125)

*ncRNA, noncoding RNA; NSCLC, non-small cell lung cancer; OSCC, oral squamous cell carcinoma; ESCC, esophageal squamous cell carcinoma; CSCC, cervical squamous cell carcinoma; PROX1, Prospero homeobox 1; FOXC2, Forkhead box C2; VEGFR-3, vascular endothelial growth factor receptor-3; VEGF-A, vascular endothelial growth factor A; VEGF-C, vascular endothelial growth factor C; DDR1, Discoidin domain receptor 1; NRP2, Neuropilin 2; Flt4, Fms-related tyrosine kinase 4; CXCL12a, Chemokine (C-X-C motif) ligand 12a; FoxO1, Forkhead box O1; LYVE-1, Lymphatic vessel endothelial hyaluronan receptor 1; WISP-1, WNT1-inducible signaling pathway protein-1; ANRIL, antisense non-coding RNA in the INK4 locus; LNMAT1, Lymph Node Metastasis Associated Transcript 1; C21orF96, Chromosome 21 open reading frame 96*.

The clinical potential function of ncRNAs as new targets has been carried out. For example, antisense oligonucleotide therapy can be applied to correct aberrant splicing (131, 132); via replacing or inhibiting ncRNAs especially miRNAs, affecting levels or functions of ncRNAs (133). Loss of MALAT1 with antisense oligonucleotide provided a potential therapeutic approach to prevent lung cancer metastasis via regulating gene expression, but not alternative splicing (134, 135). Though breakthroughs in targeted therapy have involved ncRNAs, major challenges exist with limited examples and acquired resistance.

Lymphangiogenesis has the potential to become the therapeutic target (44), since lymphatic vessels are mostly quiescent in adults and LEC identity is more plastic during adulthood than during embryo. However, some problems still need to be addressed. Interfering with tumor lymphangiogenesis can decrease or prevent lymphatic metastasis through blocking lymphatic drainage, while it may also lead to tissue fluid accumulation and cause lymphedema. Is there a target to solve both the metastasis and lymphedema? Furthermore, in a radical operation that resects primary or metastatic tumor, concurrent anti-lymphangiogenesis therapy could postpone wound healing, as lymphangiogenesis does good to inflammation in some conditions (136). That necessitates the identification of tumor lymphatics-specific markers. Recently, therapies aimed at blocking the VEGF-C/VEGF-D/VEGFR-3 signaling axis have entered clinical trials in some types of tumors, while these could not block metastasis completely (137, 138). Olmeda et al. have reported that tumors actually could induce lymphangiogenesis in distant organs rather than just SLNs before tumor cells colonization by secreting MIDKINE, and the molecular profiles and functions of LECs in distant and local lymphatic vessels might vary depending on which tissue they were in Olmeda et al. (139). As ncRNAs also have tissue-specificity expressions, their serving as a target to orchestrate lymphangiogenesis, even in more than one pathological conditions when required, is worth validation.

## Author Contributions

MC, YT, Y-JT, and XL designed the subject of the review. MC and Y-JT were involved in material collection. MC, YT, and WZ were responsible for writing of the manuscript. Y-JT, WZ, and XL were responsible for figure drawing. All authors reviewed the manuscript.

### Conflict of Interest Statement

The authors declare that the research was conducted in the absence of any commercial or financial relationships that could be construed as a potential conflict of interest.

## Abbreviations

ncRNA: non-coding RNA
PROX1: Prospero homeobox 1
BMP2: bone morphogenetic protein 2
FOXC2: Forkhead box C2
IL-1β: Interleukine-1 beta
VEGFR-3: vascular endothelial growth factor receptor-3
NSCLC: non-small cell lung cancer
VEGF-A: vascular endothelial growth factor A
VEGF-C: vascular endothelial growth factor C
NF-κB: nuclear transcription factor-κB
OSCC: oral squamous cell carcinoma
NRP2: Neuropilin 2
Flt4: Fms-related tyrosine kinase 4
CXCL12a: Chemokine (C-X-C motif) ligand 12a
FoxO1: Forkhead box O1
TNF-α: Tumor Necrosis Factor alpha
LYVE-1: Lymphatic vessel endothelial hyaluronan receptor 1
WISP-1: WNT1-inducible signaling pathway protein-1
ANRIL: antisense non-coding RNA in the INK4 locus
C21orF96: Chromosome 21 open reading frame 96.
